# Characterization of FcXTH2, a Novel Xyloglucan Endotransglycosylase/Hydrolase Enzyme of Chilean Strawberry with Hydrolase Activity

**DOI:** 10.3390/ijms21093380

**Published:** 2020-05-11

**Authors:** Luis Morales-Quintana, Dina Beltrán, Ángela Mendez-Yañez, Felipe Valenzuela-Riffo, Raúl Herrera, María Alejandra Moya-León

**Affiliations:** 1 Multidisciplinary Agroindustry Research Laboratory, Instituto de Ciencias Biomédicas, Universidad Autónoma de Chile, 3460000 Talca, Chile; 2 Functional Genomics, Biochemistry and Plant Physiology group, Instituto de Ciencias Biológicas, Universidad de Talca, 3460000 Talca, Chile; dbeltran@udec.cl (D.B.); raherre@utalca.cl (R.H.); 3 Programa de Doctorado en Ciencias Mención Ingeniería Genética Vegetal, Instituto de Ciencias Biológicas, Universidad de Talca, 3460000 Talca, Chile; Felipe.v.r.89@gmail.com

**Keywords:** xyloglucan endotransglycosylase/hydrolases, cell wall disassembly, molecular modeling, XEH activity, kinetic studies, *Fragaria chiloensis*

## Abstract

Xyloglucan endotransglycosylase/hydrolases (XTHs) are cell wall enzymes with hydrolase (XEH) and/or endotransglycosylase (XET) activities. As they are involved in the modification of the xyloglucans, a type of hemicellulose present in the cell wall, they are believed to be very important in different processes, including growth, development, and fruit ripening. Previous studies suggest that XTHs might play a key role in development and ripening of *Fragaria chiloensis* fruit, and its characterization is pending. Therefore, in order to provide a biochemical characterization of the FcXTH2 enzyme to explain its possible role in strawberry development, the molecular cloning and the heterologous expression of *FcXTH2* were performed. The recombinant FcXTH2 was active and displayed mainly XEH activity. The optimal pH and temperature are 5.5 and 37 °C, respectively. A K_M_ value of 0.029 mg mL^−1^ was determined. Additionally, its protein structural model was built through comparative modeling methodology. The model showed a typically β-jelly-roll type folding in which the catalytic motif was oriented towards the FcXTH2 central cavity. Using molecular docking, protein-ligand interactions were explored, finding better interaction with xyloglucan than with cellulose. The data provided groundwork for understanding, at a molecular level, the enzymatic mechanism of FcXTH2, an important enzyme acting during the development of the Chilean strawberry.

## 1. Introduction

Plant cell growth and morphogenesis are strongly dependent on the dynamic structure of the primary plant cell wall. Plant cell wall is composed mainly by three carbohydrate polymers: cellulose, pectins, and hemicellulose—in addition to structural proteins and ions. Several non-covalent interactions between cell wall components enables a complex structure and provides strength. Cellulose is a water-insoluble polymer and tough fiber; it is formed by β-D-glucopyranose units linked by (1,4) glycosidic bonds that form unbranched homopolysaccharides [1,2]. Hemicellulose polysaccharides consider a series of polymers that contain a backbone of β-linked sugars that can change depending of the type of hemicellulose. For example: the backbone sugar of the xylan is β-1,4-D-Xyl (xylose), that of the mannans is β-1,4-DMan (manose), in the xyloglucans the backbone is formed by β-1,4-D-Glc (glucose), while in glucomannans the backbone consists of randomly dispersed β-1,4-Glc and β-1,4-Man sugars. Additionally, these hemicellulose backbones are decorated with a variety of acetyl groups and sugars [3]. Main hemicelluloses type in dicotyledons, such as strawberry, is xyloglucans (XG) [4]. In the structure of the XG, the glucosyl residues in the backbone (named with G letter) are substituted at C (O)6 with sugars: short oligosaccharide chains of α-D-xylopyranosyl-β-D-galactopyranosyl (named with letter L), or α-D-xylopyranosyl residues (named with letter X), or less frequently with α-D-xylopyranosyl- β-D-galactopyranosyla α-L-fucopyranosyl oligosaccharide (named with letter F) [5]. In particular, XXFG and XXXG polymers are the most abundant XG described to strawberry fruit [6]. Pectins are the other main components of plant cell wall. The most abundant type of pectins includes homogalacturonan, rhamnogalacturonan I, and rhamnogalacturonan II [7]. The cell wall undergoes dynamic remodeling during plant growth and development [8,9], including fruit development and ripening [10].

The dynamics of the cell wall remodeling is governed by a number of non-enzymatic and enzymatic factors [10,11,12]. In respect to the enzymatic factor, a complete set of proteins and enzymes that participates in cell wall assembly/disassembly have been reported in various plant species. Thus, the xyloglucan endotransglycosylase/hydrolase enzymes (XTH) [13] act on the cell wall specifically on the hemicellulose fraction. XTHs have been described and classified in the Carbohydrate-Active enZYmes Database (CAZy database; http://www.cazy.org/) as family 16 of glycosyl hydrolases (GH16) and contain the significant xyloglucan endotransglycosylase C-terminus domain (C-XET), which distinguishes XTH proteins from other GH16 subfamilies [8,14,15,16,17,18].

*XTH* family genes encode enzymatic proteins that potentially display two different catalytic activities, either endotransglycosylase (XET; EC 2.4.1.207) or hydrolase (XEH; EC 3.2.1.151), or in some cases both activities [13,17,18,19,20,21,22,23,24]. The enzymes with XET activity catalyze the breakdown and molecular grafting of the β-(1-4)-xyloglucan backbone, while the XTH enzymes with XEH activity catalyze the irreversible shortening of this β-(1-4)-xyloglucan backbone [20,25]. Thus, XTH activities (XET and XEH activities) have been deeply analyzed in different biological processes and many species including dicots and monocots plants, showing that XTHs are ever-present and important for development and growth of all plants and the ripening of fruits [8,9,24,26]. As examples, XTHs (specifically AtXTH4 and AtXTH9) contribute to wood cell expansion and secondary wall formation [27]; in radiate pine, PrXTH1 is related to the bending response of stems [24]; while different authors have characterized XTHs from fruits, and several XTHs have been associated mainly with the disassembly of the cell wall during fruit ripening process [23,28,29,30,31,32,33,34].

On the other hand, the Chilean strawberry fruit (*Fragaria chiloensis*) has been described as a fruit with excellent organoleptic properties, highlighting its aroma and flavor [35]; however, the Chilean strawberry fruit has a high softening rate [35,36,37,38]. It was reported that total cell wall material decreases during fruit ripening, coincident with the loss of firmness [35,36]. Two *XTHs* genes have been identified and isolated in Chilean strawberry, isoforms *FcXTH1* and *FcXTH2*, which showed different expression profiles [37]: *FcXTH1* is expressed in ripening fruit and the encoded protein showed a strict XET activity [23]; meanwhile, *FcXTH2* shows a high expression level in vegetative tissues and a relatively low but constant expression level in developing fruit. In other strawberries, two *XTHs*, orthologous to *XTHs* of Chilean strawberry, have been described in *Fragaria x ananassa* that were associated to hemicellulose degradation [32]; and interestingly, firmer cultivars showed higher accumulation of transcripts of both genes [10,39]. In *Fragaria vesca* 26 different XTH-encoding genes were identified, and most *FvXTHs* described are expressed in ripe fruit; however, four of them (*FvXTH18* and *FvXTH20*, *FvXTH25,* and *FvXTH26*) displayed an increase in their expression pattern as the fruit ripens [40].

The type of activity of FcXTH2 has not been clarified yet; however, the length of the conserved loop 2 (around 9 or 10 residues) predicts that FcXTH2 enzyme could display XEH activity. This is an interesting issue as most XTH ripening-related proteins described so far display principally XET activity. The hydrolase activity of FcXTH2 could explain its contribution in the expansion of fruit receptacle observed during development and ripening of *F. chiloensis* fruit.

Therefore, the aim of the current study was to characterize the FcXTH2 enzyme, structurally and biochemically, and to confirm its hypothetical hydrolase activity. This was addressed by combining bioinformatic (molecular modeling and molecular docking) and biochemical strategies.

## 2. Results

### 2.1. FcXTH2 Recombinant Protein Characterization

Firstly, FcXTH2 enzyme has a molecular weight (MW) of 35.9 kDA (without the signal peptide). The complete *FcXTH2* sequence was amplified from a cDNA prepared from young leaves. Then, *FcXTH2* coding sequence was used to transform the methylotrophic *Pichia pastoris* yeast strains. The recombinant FcXTH2 protein was 12.4-fold purified by using two sequential chromatographic steps (Table 1). The recombinant FcXTH2 protein displayed mainly XEH activity and basal XET activity (Table 1) according to BCA assay and Sulová method [41]. Interesting, after each purification step, the ratio of XEH/XET specific activities increases (Table 1), supporting the notion that FcXTH2 is primarily a hydrolase enzyme.

A commercial cellulase enzyme from *Aspergillus niger* (Sigma) was employed as positive control of XEH activity, meanwhile, for XET activity, the recombinant FcXTH1 protein that displays only XET activity [23] was employed (Table 1). The purified FcXTH2 protein displays high XEH activity (Table 1). Finally, a total protein extract obtained from *F. chiloensis* fruit at the C3 stage displays both activities (Table 1).

A minor level of XET activity (~6 a.u. μg^−1^ h^−1^) was detected in *P. pastoris* strains transformed with *FcXTH2* construction, nevertheless, the level of activity is similar to the level previously described by Mendez-Yañez et al. [23] in *P. pastoris* transformed with the pPICZaA empty vector (~4 a.u. μg^−1^ h^−1^) (Table 1).

**Figure 1:**
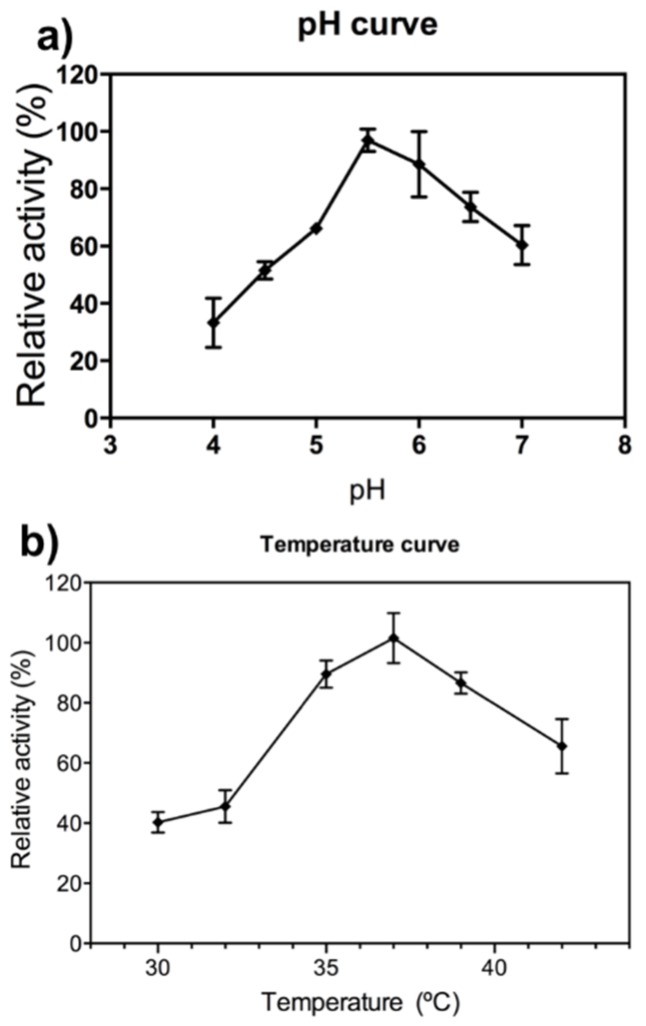
Biochemical characterization of recombinant FcXTH2 protein. (**a**) The pH dependence curve of the relative XEH activity of FcXTH2, and (**b**) temperature dependence. XEH activity was assayed as Arnal et al. (2017) [42] and expressed as percentage of maximum activity ± SE of four replicates.

As part of the characterization of FcXTH2 enzyme, its temperature and pH optimum were analyzed (Figure 1a). The purified FcXTH2 protein exhibited a bell-shaped pH profile, with an optimum pH around 5.5. Furthermore, a rapid loss of XEH activity was detected below pH 5; only 29% of activity remains at pH 4. In addition, the optimum temperature for XEH activity was 37 °C for FcXTH2 recombinant enzyme (Figure 1b).

The kinetic properties of the purified recombinant FcXTH2 enzyme were studied and the principal parameters are showed in the Table 2. K_M_ and V_max_ values were obtained from Michaelis−Menten saturation curves, and values calculated from nonlinear least-squares data fitting (NLSF) in GraphPad Prism 6. With values of 0.029 mg mL^−1^ and a 4.1 × 10^5^ μg^−1^ mL^−1^ s^−1^ to K_M_ and V_max_ respectively (Table 2).

### 2.2. FcXTH1 Structural Model

**Figure 2:**
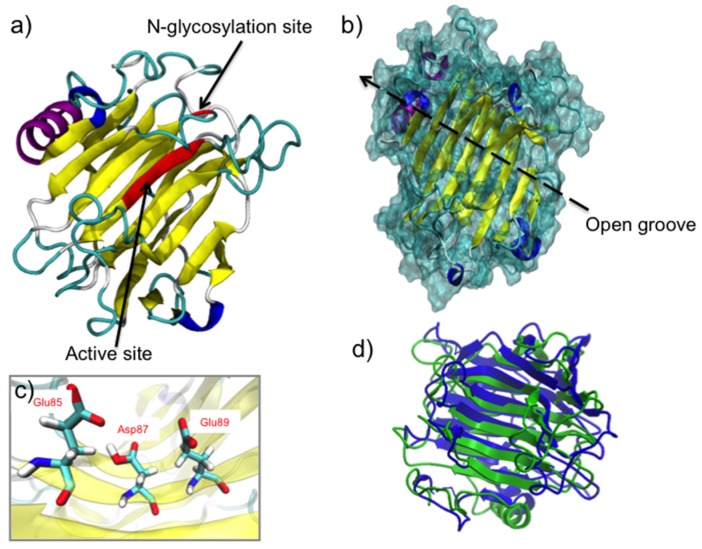
FcXTH2 protein model. (**a**) The three-dimensional (3D) model of FcXTH2 was built by comparative modeling using TmNXG1 (Protein Data Bank (PDB) code: 2UWA) as template. The active site (β-sheet 7) and the N-glycosylation site are highlighted in red. (**b**) FcXTH2 in surface representation; the location of the groove is indicated as a black line. (**c**) Zoom of the active site showing the relevant residues (in licorice view). (**d**) Structural superposition of the FcXTH2 model and the 2UWA structure. The cartoon structure of the template is colored in blue and that of FcXTH2 in green. The root mean square deviation (RMSD) value of 8.6 Å.

The three-dimensional (3D) structure of FcXTH2 is not known. We used a series of in silico tools to obtain insights into the molecular mechanism that could explain the interaction of FcXTH2 and xyloglucans as substrate. FcXTH2 was built based on the sequence alignment between FcXTH2 and TmNXG1 (Protein Data Bank (PDB) code: 2UWA) as template: the sequence identity was 44.5%. Two further optimization steps were performed to obtain the correct model for FcXTH2. First, an energy minimization procedure, and after that, a short molecular dynamics simulation provided the final structural model for FcXTH2 (Figure 2a).

The geometric and energetic evaluation of the model was performed to validate the quality of FcXTH2 structural model. The root mean square deviation (RMSD) value calculated for the backbone of FcXTH2 and its template was 8.6 Å (Figure 2d). The stereochemical quality of the FcXTH2 model structure was analyzed using the PROCHECK program and Ramachandran plots. The analysis showed all amino acid residues with φ/ψ angles at favored regions (including the most favorable regions, additional allowed regions, and generously allowed regions), but the most relevant issue is that no residues were found in bad conformation, indicating a good stereochemical quality (Figure S1a). A MDS of 2 ns showed that the protein model has a RMSD value around 1.2 Å, meaning that the modeled protein structure was stable (data not shown). Finally, the FcXTH2 model showed a Z-score of −5.7 according to ProSA-Web (Figure S1b), which was close to −6.25 value obtained for the template. Finally, the Verify3D program showed that 94.1% of the residues had a score ≥ 0.2, and 100% of the residues averaged a 3D–1D ratio score ≥ 0.0, indicative of favorable scores for the FcXTH2 protein model (Figure S1c). Consequently, the final structure of FcXTH2 protein was accepted for subsequent analysis.

The FcXTH2 structural model consists of a β-jelly-roll type structure (Figure 2a) in which several antiparallel β-sheets form a curvature in the structure (colored in yellow in the Figure 2a), placing the conserved catalytic domain in the middle area of this open groove formed by the curvature, and oriented to the solvent area (Figure 2a, red β-sheet). The folding topology consists of two antiparallel β faces, one convex and one concave, which forms a β turn with an open groove for substrate binding (Figure 2b), similar to the template structure. An analysis of the FcXTH2 model at secondary structure level showed that the enzyme consists of 1 α-helix, 2 3_10_ helices and 15 β-sheets, all of them connected by 20 loops (Figure 2a). The catalytic domain is composed by Glu85, Asp87, and Glu89 residues and is located in β-sheet 7, between loops 7 and 8 (Figure 2c). Additionally, the FcXTH2 showed a N-glycosylation site that is located far to the active site (around 15 Å of the active site) and oriented on the edge of the opposite face (Figure 2a). In the primary sequence of the protein, the N-glycosylation site is spaced by 17 residues from the catalytic triad (Figure S2).

Different authors mentioned previously that the length of loop 2 is important for the balance of XET and XEH activities [13,23,29,43]. When the structure of FcXTH2 was analyzed, it was observed that the extension of loop 2 includes nine amino acid residues and, therefore, is similar in length and in orientation to TmNXG1 (ten residues), and shows XEH activity. On the other hand, the loop is five residues longer than loop 2 of PttXET16A (Figure S3) that shows strict XET activity [13].

### 2.3. Protein-Ligand Interaction Analysis

FcXTH2 structural model and three different octasaccharides as ligands were used to evaluate the protein-ligand interactions by automatic docking studies. Table 3 shows that all interaction values are negative, which indicates that the favorable binding energies were obtained between FcXTH2 and the different ligands. Nevertheless, the interaction of FcXTH2 with XXXGXXXG was highly favored (−9.2 kcal mol^−1^), and a weaker binding interaction was found between the protein and cellodextrin 8-mer or XXFGXXFG as ligands (around −7 kcal mol^−1^) (Table 3).

**Figure 3:**
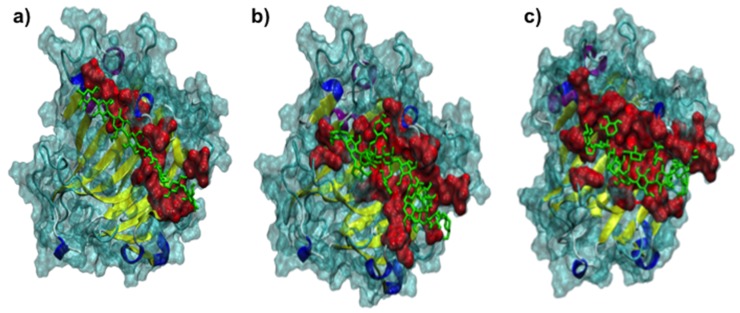
Protein–ligand interaction mode of FcXTH2. A general view of the position of ligands within the open groove of FcXTH2 protein model: (**a**) cellulose, (**b**) XXXGXXXG, and (**c**) XXFGXXFG. Residues involved in protein-ligand interaction in each protein are shown in red; the ligands are shown in green.

In general, it can be observed that the different octasaccharides are positioned along the open groove of the protein (Figure 3). Interestingly, only XXXGXXXG hemicellulose octasaccharide locates close to (≤ 3 Å) two of the catalytic residues (Glu85, Asp87) being able to interact with them (Table 4). In addition, XXXGXXXG locates close to other 17 amino acid residues from FcXTH2, being able to interact with them. On the other hand, neither cellodextrin 8-mer nor XXFGXXFG locate close to the catalytic residues. In addition, both the cellulose type substrate and XXFGXXFG could interact with a lower number of residues (11 and 12 residues, respectively) than XXXGXXXG (Table 4). Important differences in the interaction of XXXGXXXG and XXFGXXFG with FcXTH2 are observed, which suggests that the ramifications of XXFGXXFG polymer could interfere with the correct orientation of the ligand in the FcXTH2 open groove (Table 4).

## 3. Discussion

Several biochemical and biophysical modifications take place during the fruit ripening process differentiation, which contribute to encourage its seed dispersal and/or to the formation of an attractive fruit for the final consumer [44]. During fruit development and ripening, the cell wall structure changes irreversibly in a process that involves two phases (cell division and expansion). In fruits such as tomatoes, these phases are very marked [45], meanwhile, in strawberries, it is difficult to establish a functional separation of these phases, as cell division and expansion takes place in parallel [46].

The expression of several genes is modulated during development and ripening in strawberry fruit, particularly xyloglucan endotransglycosylase/hydrolase (XTH) [37,47,48,49]. XTH enzymes have an important role in cell wall loosening through the modification of the β-(1-4)-xyloglucan backbone [50,51]. For this reason, to understand xyloglucan modifications taking place in *F. chiloensis* fruit, XTH enzymes were biochemically and structurally characterized. FcXTH1 was previously characterized [23] and displayed transglycosylase activity.

### 3.1. FcXTH2 Mainly Has XEH Activity

The catalytic mechanism of XTH-related proteins is described as a double displacement/retaining mechanism. In the first step, the enzyme breaks a donor xyloglucan that liberates small xyloglucans with a reducing terminal end, and in a second step, depending on the case of XET activity [15], or in the case of XEH activity, the acceptor is a water molecule [20]. Thus, XEH activity promotes a rapid cell wall breaking and yields irreversible xyloglucan chain shortening [20]. In this work, sensitive colorimetric assays were employed to detect XET and/or hydrolytic XEH activities of a recombinant FcXTH2 enzyme expressed in *P. pastoris* and purified from the yeast culture medium. Recombinant FcXTH2 protein has principally XEH activity; the basal value of XET activity determined in the enzyme is similar to the activity of yeasts transformed with the empty vector (Table 1). The specificity of hydrolase activity of FcXTH2 is similar to TmNXG1 [52] and to AtXTH33 [25], which was used as a template to build the comparative model of FcXTH2.

Several authors have argued against the in vivo existence of these two activities [53], while others consider that all XTHs could present both (XET and XEH) in vivo activities, albeit at different levels [52]. Thus, there are some XTHs that show in vitro XEH activity but not XET activity, such as those isolated from azuki bean (*Vigna angularis*) and Nasturtium (TmXTH1 or TmNGX1, *Tropaeolum majus* L.) [43,54]. In contrast, some XTHs have a strong XET activity but also presents a low XEH activity, for example, the recombinant SlXTH5 protein from tomato fruit [52]. Shi et al. (2015) [25] recently showed that AtXTH15 had high in vitro XET activity but little XEH activity, whereas AtXTH33 had only slight in vitro XET activity but high XEH activity (both enzymes expressed in *P. pastoris*) with XEH:XET activity ratio of > 5000:1 [55].

On the other hand, Opazo et al. (2010) [37] previously reported the total XTH activity for different *F. chiloensis* fruit stages. The highest XET and XEH activities were observed at the turning stage (or named C3 stage), when the fruit is still growing and under intensive softening. The contribution of both FcXTH1 (mainly XET activity) and FcXTH2 (with XEH activity) is crucial for cell wall disassembling.

For XTH enzymes, the phylogenetic classification tree has been reported as a good predictor of their activity, being possible to discriminate between both activities (XEH and / or XET activity) [20,23,25,37,40,56]. Thus, *XTH* genes were originally divided into three major groups [19,52] that were named as Group: I/II, III, and an ancestral group. With the expansion of *XTH* gene studies, a more detailed clade and subclade grouping has been established and nowadays the group III was subdivided into IIIA and IIIB [16,34,37,40,52]. XTH enzymes from group I/II have been described as strict XET [20,25,56], while members of group III-A group have been shown to display XEH activity [52,53,54,55,57,58]. FcXTH2 belongs to group III-A [37], and coincident with its phylogeny, it has XEH activity (Table 1).

The biochemical characterization of FcXTH2 allows the determination of optimum pH and temperature values for activity, values that are similar to those reported for FcXTH1 [16], and other XTHs previously described such as AtXTH3 (XET activity, optimum pH between 4.5 to 5.5 and 30 °C) [17]. The K_M_ value determined for FcXTH2 enzyme against XG heptasaccharide was 0.029 mg mL^−1^. This value is similar to the K_M_ reported for FcXTH1 enzyme (0.06 mg mL^−1^ for the glycosylated form; 0.162 mg mL^−1^ for deglycosylated enzyme) [23].

### 3.2. The 3D Structure of FcXTH2 and Protein-Ligand Interaction Based on Comparative Modeling and Docking Studies

FcXTH2 was structurally characterized using molecular modeling. The structure showed the characteristic fold of proteins belonging to the GH16 family, which is a β-sandwich curvature generated by β-sheets oriented in antiparallel form [13,15,23,26]. Additionally, we observed that the residues of the catalytic domain (ExDxE motif) were oriented towards the solvent area in the middle of the open groove formed (in β7) to allow the binding of putative substrates (Figure 3) [13,15,23,59].

On the other hand, the FcXTH2 model was built at its optimal enzymatic activity (pH 5.5), and at this pH, the catalytic residues (Asp81 and Glu83) are protonated, meanwhile Glu79 is not. This conformation is coherent with the nucleophile attack required for enzyme activity proposed by Baumann et al. (2007) [43], and deduced from the crystallographic structure [13]. It is also similar to the protein model of PrXTH1 described by Valenzuela et al. (2014) [26] and FcXTH1 described by Mendez-Yañez et al. (2017) [23]. Nevertheless, this conformation differs from the one described earlier by Mark et al. (2011) [60] in relation to Glu83 residue. The authors indicate that the catalysis is optimal when the ‘helper residue’ (Asp87 in PttXET16-34, equivalent to Asp81 in FcXTH2) is protonated, the catalytic nucleophile residue (Glu85 in PttXET16-34, equivalent to Glu79 in FcXTH2) is deprotonated for nucleophilic attack on the substrate, and the general acid/base residue (Glu89 in PttXET16-34, equivalent to Glu83 in FcXTH2), is in conjugated acid form, to align the nucleophile and deliver a proton to the departing sugar [60].

Molecular docking studies indicate different affinity energy values for the series of ligands tested for their interaction with FcXTH2, XXXGXXXG being the best ligand (Table 3). These results are comparable to those published for other XTHs, such as AtXTH15, with better preference for XXXG as substrate than XGOs (Shi et al., 2015), and similar to the data obtained for FcXTH1 by the same in silico approach [23] where the FcXTH1-cellulose interaction was around −7 kcal mol^−1^.

## 4. Methods

### 4.1. Plant Material 

*F. chiloensis* fruit with white receptacle and red achenes, corresponding to C3 stage [35,36], were used to determine the total XET and XEH activities present in the fruit. The fruit was harvested from plants grown in a commercial field in Purén, The Araucania Region, Chile (latitude 38° 040S; longitude 73° 140 W).

### 4.2. Extraction of Proteins

Fruit samples at C3 stage were employed to quantify total enzyme activity according to Opazo et al. (2010) [37]. For that, 10 grams were homogenized in extraction buffer (40 mmol L^−1^ sodium acetate, 13 mmol L^−1^ EDTA, 10 mmol L^−1^ β-mercaptoethanol, 1% *w*/*v* PVP, 1 mol L^−1^ NaCl, pH 5.0) and grounded in a mill (MM301, Retsch, Haan, Germany). The homogenate was incubated at 4 °C for 18 h in agitation, and centrifuged at 13,000 rpm for 30 min. The supernatant was filtered, and the proteins were precipitated at 4 °C with 90% of ammonium sulfate, and centrifuged at 13,000 rpm for 15 min. The pellet was suspended in sodium acetate (40 mmol L^−1^ at pH 6.0) and the suspended pellet was desalted using a Sephadex G-25 column (PD-10, GE Healthcare, IL, USA). The final desalted extract was employed for the assay of XET and XEH activities (Section 4.4) and protein concentration analysis by the Bradford method [61].

### 4.3. Protein Production and Purification

The cDNA sequence of *FcXTH2* was previously identified and reported (GeneBank accession number GQ280283) by Opazo et al. (2010) [37]. After the removal of its signal peptide and the stop codon, the coding sequence of the *FcXTH2* gene was amplified by RT-PCR using the primers forward 5′-CTGCAGCCAAAACTCTACCAATC-3′ and reverse 5′-TCTAGAAACAGAAGAAGTGTCTGCT-3′ to introduce at the 5′ and 3′ ends the *PstI* and *XbaI* restriction sites, respectively. cDNA from *F. chiloensis* young leaves was obtained with a first strand cDNA synthesis kit (Thermo Scientific, Waltham, MA, USA), and it was used as PCR template. Following manufacturer guidelines, *FcXTH2* coding sequence was cloned into pJET1.2 vector (CloneJET PCR Cloning Kit^®^, Fermentas of Thermo Fisher Scientific; Waltham, MA, USA). *Escherichia coli* colonies were checked by PCR and positive colonies were used for plasmid extraction (GeneJET^®^ Plasmid Miniprep Kit, Thermo Fisher Scientific; Waltham, MA, USA) according to the manufacturer’s protocol. The *FcXTH2* gene was cloned in *Pichia pastoris* (strain X-33) including a His tag sequence at the 3’ end to facilitate the purification procedure. The production of recombinant FcXTH2 protein was carried out according to the method employed by Mendez-Yañez et al. (2017) [23] and previously standardized in the laboratory. The purification of the recombinant FcXTH2 protein was performed by the use of two different chromatographic criteria: first, an anion exchanger, and second, by the use of an affinity chromatography. A carboxymethyl cellulose resin (CMC, Sigma-Aldrish, MO, USA) was used as a cation exchanger chromatography in 0.05 M sodium phosphate buffer (pH 6.0), and elution of protein was accomplished in the same buffer containing 0.5 M NaCl. After desalting through PD-10 columns (Sephadex™ G-25 Medium, SigmaAldrich of Merck KGaA, Darmstadt, German ), a Talon Metal Affinity column (BD Biosciences; San Jose, CA, USA) was employed in the presence of 0.05 M sodium phosphate buffer (equilibrium buffer at pH 7.4, washing buffer at pH 6.8), and elution of bound protein with buffer containing 0.025 M imidazole (pH 6.0), according to the method employed by Mendez-Yañez et al. (2017) [23] and previously standardized in the laboratory.

### 4.4. XET and XEH Assays

XET activity was assayed by the colorimetric method described previously by Sulová et al. (1995) [41] and implemented previously in the laboratory previously by Mendez-Yañez et al. (2017) [23]. The Sulová method for XET activity is based in the ability of long XGs to bind iodine: the transglycosylation activity causes a reduction in size of XGs that loses the ability to bind the dye. Thus, the XET activity causes the reduction in molecular weight of XGs by transglycosylation of portions of the XG donor to low relative molecular mass (low-*Mr*) oligosaccharide acceptors whereby no net formation of reducing ends takes place. Thus, the XET activity was assayed by following changes in absorbance at 620 nm in an Epoch 2 microplate spectrophotometer (BioTek Instruments, Winooski, VT, USA). The XG-transglycosylating (XET) activity was expressed as arbitrary units (a.u.) ug protein^-1^ h^−1^ as described by Sulová et al., (1995) [41]. 

Recently, Arnal et al., (2017) [42] described a sensitive assay to determine the XEH activity of XTH enzymes using the BCA (disodium 2,20-bicinchoninic acid) method for reducing sugars at small-volume scale. As XEH activity degrades xyloglucan by hydrolysis, it is possible to follow the production of reducing sugars. The increment in reducing sugars was evaluated through changes in absorbance at 562 nm in an Epoch 2 microplate spectrophotometer (BioTek Instruments, Winooski, VT, USA), using glucose (0–50 μM) was used as standard.

For each measurement, four replicates were employed and values correspond to means ± SE. Purified FcXTH1 protein expressed in *P. pastoris* [23] was used as XET positive control, and temperature-inactivated FcXTH2 enzyme (incubation at 100 °C for 10 min) was used as negative control.

### 4.5. pH and Temperature Dependence and Kinetic Characterization

To determine optimum assay conditions, pH was modified from 4 to 7, and the temperature from 30 to 42 °C similar to previously described [23]. XEH activity was expressed in relative terms as percentage (%) of maximum activity. 

To determine kinetic parameters Michaelis−Menten saturation curves were built using substrate concentrations between 0.0 to 0.30 mg mL^−1^ XG-subunit oligosaccharides (XGOs) under standard assay conditions. The K_M_ and V_max_ values were calculated from nonlinear least-squares data fitting (NLSF) in GraphPad Prism 6 (GraphPad Software Inc., San Diego, CA, USA). Determinations were performed in quadruplicates and expressed as mean ± SE.

### 4.6. Obtaining the 3D Structure of FcXTH2 by Homology Modeling

The 3D model of FcXTH2 protein was built by comparative modeling based on high-resolution crystal structures of homologous proteins according to Morales-Quintana et al. (2011) [62]. In short, and specifically with respect to FcXTH2, the crystal structure of a *Nasturtium* seedling xyloglucanase isoform named TmNXG1 (PDB code: 2UWA) [43] was selected as template among the structures available at the Brookhaven Protein Data Bank (PDB) as template. MODELLER 9v14 software [63] (http://salilab.org/modeller/) was used to obtain fifty comparative models. Protein hydrogen atoms were added according to environment at pH 5.5 using the HBUILD module of CHARMM version c31b1 [64] based in the Mendez-Yañez et al. (2017) [23] methodology. Equilibration and evaluation methods were performed according to the method employed by Morales-Quintana et al. (2011) [62], using Nano Molecular Dynamics (NAMD 2.11) software [65] to the equilibrate the model, meanwhile ProSA-Web [66,67] and PROCHECK v.3.5.4 [68] were employed to evaluate the quality of the model.

### 4.7. Protein-Ligand Interactions

Docking studies were performed to predict the putative binding of different ligands to the FcXTH2 protein model. Two hemicellulose octasaccharides (XXXGXXXG and XXFGXXFG) were tested, and cellodextrin 8-mer that resembles a water-soluble cellulose molecule was employed as negative control. The three ligands were obtained from Gaete-Eastman et al. (2015) [69]. AutoDock Vina v1 program (http://vina.scripps.edu/) [70] was used for the analysis. For calculations a grid size of 65 Å × 68 Å × 125 Å centered at the interface with 0.375 Å spacing was used, that includes all protein surface. A Lamarckian genetic algorithm was used as a search method, and the rest of the parameters were set with their default values.

## 5. Conclusions

FcXTH2 recombinant enzyme displays mainly XEH activity, and after each purification step, the ratio of specific activities XEH/XET increases, supporting the notion that FcXTH2 is primarily a XEH. The acid growth hypothesis states that upon acidification of the cell wall, it becomes more extensible. In this sense, and according to the optimal pH described for FcXTH2 activity (optimal activity at pH 5.5), it is possible that FcXTH2 enzyme can be involved in the fruit growth. In this sense, FcXTH2 is proposed as an important enzyme in the fruit development process, specifically in the fruit growth process. We support this proposal; however, it is important to consider that the activity of a recombinant enzyme tested in vitro could not be the same in in vivo or in plant conditions. Therefore, the idea of FcXTH2 being involved in the acid-growth is only an assumption. Additionally, the different ligands interact with FcXTH2 at the open groove, and the best binding was obtained with the xyloglucan XXXGXXXG, which was also described as one of the most abundant XG types present in strawberries.

## Figures and Tables

**Table 1:** Purification of recombinant FcXTH2 protein expressed in *P. pastoris.*

Fraction	Volume (mL)	Protein (mg/mL)	XET Activity (a.u. μg^−1^ h^−1^)	XEH Activity (a.u. μg^−1^ h^−1^)	Purification Fold (XEH Act)
Concentrated crude extract	10	6.82	6.47 ± 1.6	1.23 ± 2.3	1
Anion exchange chromatography Eluted protein (0.5 M NaCl)	10	5.70	2.56 ± 2.0	5.67 ± 2.0	4.61
Affinity chromatography Eluted purified protein (25 mM imidazole)	2	3.91	2.71 ± 1.1	15.30 ± 1.5	12.43
Cellulase from *A. niger* ^1^	-	-	0.0 ± 1.1	65.7 ± 4.0	-
Recombinant FcXTH1 protein from *F. chiloensis* ^2^	-	-	63.45 ± 4.0	0.0	-
Fruit extract of *F. chiloensis* ^3^	-	-	75.0 ± 3.2	13.82 ± 0.7	-
Empty vector pPICZαA ^4^	-	-	4.24 ± 2.6	7.4 ± 2.7	-

Protein extracts used as controls: (1) cellulase from *A. niger* used as control for hydrolase (XEH) activity; (2) recombinant FcXTH1 used as control for strict endotransglycosylase (XET) activity [23]; (3) a protein extract prepared from *F. chiloensis* fruit, described to display both XET and XEH activities [23,37]; (4) extract obtained from *P. pastoris* transformed with the empty vector pPICZαA, used for XET/XEH basal activities. XET activity was assayed as Sulová et al. (1995) [41] at pH 5.5. XEH activity was assayed as Arnal et al. (2017) [42] at pH 5.5. Values correspond to mean ± SE of four replicates.

**Table 2:** Kinetic parameters of purified FcXTH2 protein. XEH activity was assayed as Arnal et al. (2017) [42].

Protein Name	K_M_^#^ (mg mL^−1^)	V_max_ (μg mL^−1^ s^−1^)	k_cat_^#^ (s^−1^)	k_cat_ * K_M_^−1^ ((mg/mL)^−1^ s^−1^)
FcXTH2	0.029 ± 0.003	4.1 × 10^5^	8.21 × 10^3^	2.83 × 10^2^

# Values were rounded to the third decimal point.

**Table 3:** Affinity energy of FcXTH2 protein model with three different octasaccharides as putative ligands. Determinations were performed by docking simulations using AutoDock Vina.

Substrates	Affinity Energy ( kcal mol^−^^1^)
XXXGXXXG	−9.2 ± 0.9
XXFGXXFG	−7.1 ± 0.8
cellodextrin 8-mer	−7.2 ± 1.1

**Table 4:** List of residues that are located at ≤ 3Å in the complex between FcXTH2 and each ligand tested. The catalytic residues are highlighted in bold.

FcXTH2 in Complex with		
Cellodextrin 8-mer	XXFGXXFG	XXXGXXXG
Asn13	Asn13	Asn13
Val43	Val43	Val43
Gln45	Gln45	Ala70
Ser74	Leu47	Met78
Asp77	Tyr73	Pro80
His83	His83	His83
Gly109	Asn104	Glu85
Thr111	Tyr106	Asp87
Thr113	Gly109	Gln102
Ser169	Arg118	Asn104
Tyr171	Trp180	Tyr106
	Ile213	Thr111
		Gly114
		Glu116
		Arg118
		Ser169
		Thr173
		Trp180
		Arg259

## Supplementary Materials

The following are available online at https://www.mdpi.com/1422-0067/21/9/3380/s1. Figure S1: Evaluation of the model quality of FcXTH2. (A) Ramachandran plot by PROCHECK software, (B) Energetic evaluation using ProSA software, (C) and Verify3D graph of the FcXTH2 before (blue line) and after (red line) of Molecular Dynamic simulation, Figure S2: Distance of the N-glycosylation site (in yellow box) of the active site of different XTH including FcXTH2 (red box), Figure S3: Alignment of the deduced full-length amino acid sequence of FcXTH2 with TmNXG1and PttXET16A. TmNXG1 showed in vitro XEH activity but not XET activity. Meanwhile, PttXET16A showed in vitro only XET activity.

## Abbreviations

XET	Xyloglucan endotransglycosylase activity
XEH	Xyloglucan hydrolase activity
FaXTH2	Xyloglucan endotransglycosylase/hydrolases from Fragaria chiloensis isoform 2.
XG	Xyloglucan
BCA	Disodium 2,20-bicinchoninic acid
