# Supplementary material for: Characterization of FcXTH2, a Novel Xyloglucan Endotransglycosylase/Hydrolase Enzyme of Chilean Strawberry with Hydrolase Activity
Source: Int J Mol Sci. 2020 May 11;21(9):3380. doi: 10.3390/ijms21093380 (PMC7247008; doi:10.3390/ijms21093380)
Supplement: Supplementary file 1 [file ijms-21-03380-s001.pdf]

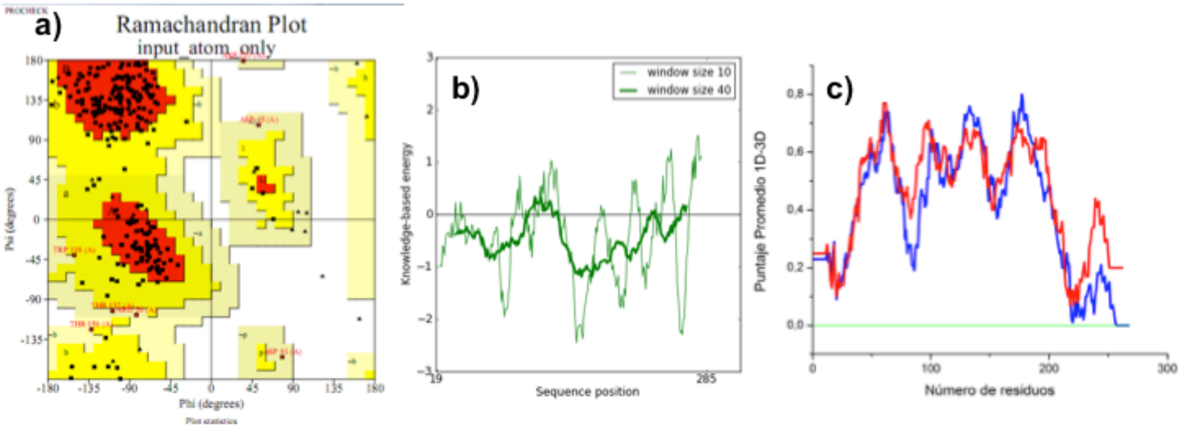

Figure S1. Evaluation of the model quality of FcXTH2. (A) Ramachandran plot by PROCHECK software, (B) Energetic evaluation using ProSA software, (C) and Verify3D graph of the FcXTH2 before (blue line) and after (red line) of Molecular Dynamic simulation**.**

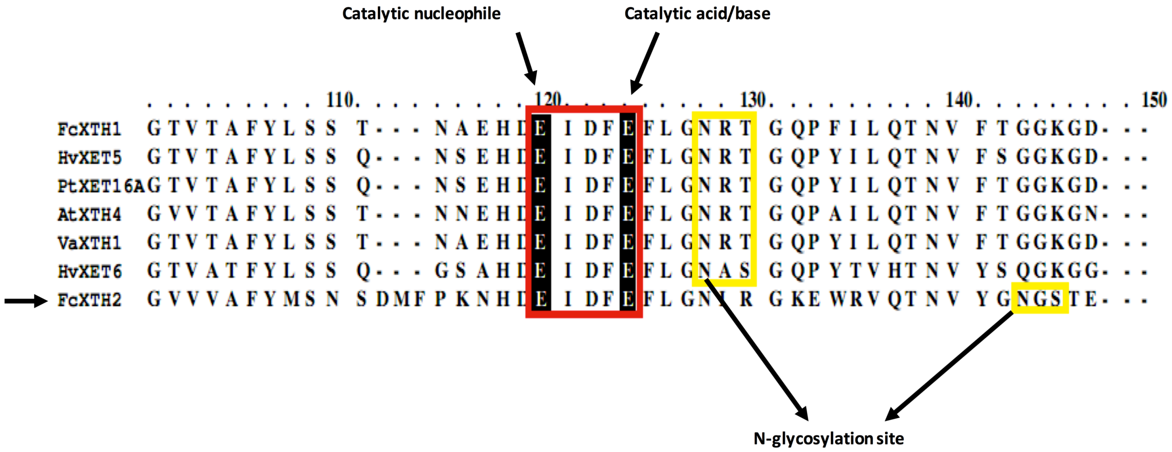

Figure S2. Distance of the N-glycosylation site (in yellow box) of the active site of different XTH including FcXTH2 (red box).

```
1UMZ        -----XETAAFAALRKPVDVAFGRNYVPTWAFDHIKYFNGGNEIQLHLDKYTGTGFQSKG    55
2UWA        AYVQGPPSPGYYPSSQITSLGFDQGYTNLWGPQHQRV--DQGSLTIWLDSTSGSGFKSIN    58
FcXTH2      --------------KTLPIMSFDEGYNKLFGDDNLMILKDGKSVHLTLDERTGSGFVSQD    46
                               :.*...*   :. ::     .  .: : **. :*:** * .

1UMZ        SYLFGHFSMQMKLVPGDSAGTVTAFYLSSQN---SEHDEIDFEFLGNRTGQPYILQTNVF    112
2UWA        RYRSGYFGANIKLQSGYTAGVITSFYLSNNQDYPGKHDEIDIEFLGTIPGKPYTLQTNVF    118
FcXTH2      LYIHGFFSASIKLPTDYTAGVVVAFYMSNSDMFPKNHDEIDFEFLGNIRGKEWRVQTNVY    106
             *  *.*. .:**  . :**.:.:**:*..:    :*****:****.  *: : :****:

1UMZ        TGGKGD-----REQRIYLWFDPTKEFHYYSVLWNMYMIVFLVDDVPIRVFKNCKDLGVKF    167
2UWA        IEGSGDYNIIGREMRIHLWFDPTQDYHNYAIYWTPSEIIFFVDDVPIRRYPRK--SDATF    176
FcXTH2      --GNGSTE-TGREERYNLWFDPSEDYHQYSILWTDSQIIFYVDNIPIRVVKKSESMGGDY    163
              *.*: . ** *  *****::::* *:: *.   *:* **::***   .    .  :
               Loop2

1UMZ        PFNQPMKIYSSLWNADDWATRGGLEKTDWSKAPFIASYRSFHIDGCEASVEAK---FCAT    224
2UWA        PL-RPLWVYGSVWDASSWATENGKYKADYRYQPFVGKYEDFKLGSCTVEAASS----CNP    231
FcXTH2      PS-KPMSLYATIWDGSGWATDGGKYRVNYKYAPFRAEFSDLVLHGCAVDPIEQVSKKCDK    222
            *  :*: :*.::*:...*** .*  :.::   ** ..: .: : .* ..   .    *

1UMZ        QGARWWDQKEFQDLDAFQYRRLSWVRQKYTIYNYCTDRSRYPSMPPECKRDRDI------    278
2UWA        ASVSPYG---Q--LSQQQVAAMEWVQKNYMVYNYCDDPTRDHTLTPEC------------    274
FcXTH2      TQGSEYI---PTGVTRLQRMRMESFRKRQMTYSYCYDRIRYKVAPPECVITPQEAERLRV    279
                 :       :   *   :. .::.   *.** *  *     ***

1UMZ        --------------------------------      278
2UWA        --------------------------------      274
FcXTH2      FDPVTFGHGHRRQRKHHHRSSSRASVADTSSV      311
```

Figure S3. Alignment of the deduced full-length amino acid sequence of FcXTH2 with TmNXG1and PttXET16A. TmNXG1 showed *in vitro* XEH activity but not XET activity. Meanwhile, PttXET16A showed in vitro only XET activity.
